# Soluble CD14 and Lipopolysaccharide-Binding Protein Are Not Superior to Soluble CD25 as Biomarkers for Sarcoidosis

**DOI:** 10.3390/diagnostics16071018

**Published:** 2026-03-28

**Authors:** Sabine Ammann, Pedro Marques-Vidal, Matthieu Perreau, Camillo Ribi

**Affiliations:** 1 Service of Immunology and Allergy, Lausanne University Hospital, University of Lausanne, 1011 Lausanne, Switzerland; matthieu.perreau@chuv.ch (M.P.); camillo.ribi@chuv.ch (C.R.); 2 Department of Medicine, Internal Medicine, Lausanne University Hospital, University of Lausanne, 1011 Lausanne, Switzerland; pedro-manuel.marques-vidal@chuv.ch

**Keywords:** sarcoidosis, soluble CD14, lipopolysaccharide-binding protein, soluble interleukin-2 receptor, angiotensin-converting enzyme, biomarkers

## Abstract

**Background/Objectives**: Sarcoidosis is a granulomatous disease with no widely accepted circulating biomarkers for routine diagnostics. Soluble CD14 (sCD14) and lipopolysaccharide-binding protein (LBP), identified through extracellular vesicle proteomics, have been proposed as candidates. We aimed to compare the diagnostic accuracy of serum sCD14 and LBP with the established biomarker soluble interleukin-2 receptor alpha chain (sCD25). **Methods**: A matched case–control study included 46 newly diagnosed, untreated sarcoidosis patients and 46 age- and sex-matched healthy controls. Serum sCD14, sCD25, and LBP were quantified by ELISA. BMI was included as a covariate in multivariable logistic regression. Diagnostic performance was evaluated by ROC analysis and stepwise AIC model selection. Longitudinal biomarker dynamics were assessed in 32 patients under treatment. **Results**: sCD25 demonstrated superior diagnostic discrimination (AUC 0.92, 95% CI 0.87–0.98), compared with LBP (AUC 0.71, 95% CI 0.60–0.82) and sCD14 (AUC 0.61, 95% CI 0.49–0.73). In multivariate analysis, only sCD25 (OR per +100 pg/mL: 1.53; *p* < 0.001) remained an independent predictor of sarcoidosis. Neither LBP nor sCD14 improved model fit. All biomarkers significantly decreased following therapy. **Conclusions**: Among routinely measurable serum markers, sCD25 outperformed sCD14 and LBP in sarcoidosis diagnosis. Further studies should explore immunometabolic interactions to refine diagnostic algorithms.

## 1. Introduction

Sarcoidosis is a multisystemic inflammatory disease of unknown etiology, characterized by the formation of non-caseating granulomas. Its pathogenesis likely involves a complex interplay between genetic susceptibility and environmental triggers, including exposure to infectious agents. Granulomas consist of T lymphocytes, activated mononuclear phagocytes, and multinucleated giant cells. The inflammatory process may infiltrate multiple organs. Clinical manifestations are highly variable [1,2].

Biomarkers in sarcoidosis are valuable for diagnosis and may also indicate disease activity and treatment response. Serum angiotensin-converting enzyme (ACE) is a widely used biomarker and appears to correlate with total granuloma burden. However, its diagnostic performance is limited, with sensitivity and specificity of 60% and 93%, respectively [3]. ACE reflects disease activity only in patients with elevated baseline levels and is influenced by ACE inhibitor therapy. Furthermore, ACE concentrations are affected by I/D polymorphisms of the ACE gene, which independently influence baseline activity [4,5]. The soluble interleukin-2 receptor alpha chain (sIL-2R or sCD25) is a marker of T-cell activation, demonstrating higher sensitivity (88%) with good specificity (87%). sCD25 typically decreases during treatment and remission, suggesting utility for monitoring disease activity [6]. Nevertheless, its diagnostic value remains controversial, as elevated levels are not specific to sarcoidosis [7,8,9,10]. To date, no single biomarker combines high sensitivity and specificity, underscoring the need for improved diagnostic tools.

Soluble CD14 (sCD14) and lipopolysaccharide-binding protein (LBP) were identified as potential sarcoidosis biomarkers through proteomic analysis of serum extracellular vesicles by Futami et al. [11]. Notably, their expression was not increased in control groups with other inflammatory lung diseases such as COPD, asthma or lung fibrosis, suggesting good specificity for sarcoidosis.

CD14 is a myeloid differentiation antigen primarily expressed on monocytes and macrophages. During inflammation or infection, activation of these cells leads to shedding of CD14, resulting in elevated levels of its soluble form (sCD14) [12]. A single study in pulmonary sarcoidosis reported increased CD14 expression on alveolar macrophages and higher serum sCD14 concentrations, both correlating with impaired lung function, indicating a possible link to disease severity [13].

LBP is an acute-phase protein that binds lipopolysaccharide (LPS) and facilitates its transfer to CD14, triggering Toll-like receptor (TLR) signaling. This cascade promotes cytokine production, co-stimulation, and phagocyte maturation. Elevation in LBP suggests recent or previous exposure to microbial or environmental antigens, which are believed to induce granulomatous inflammation in genetically predisposed individuals [14].

However, evidence supporting the clinical utility of sCD14 and LBP in sarcoidosis remains limited. To date, their diagnostic performance has not been independently replicated, nor directly compared with established circulating biomarkers within the same patient population.

The aim of the present study was to evaluate the diagnostic performance of soluble sCD14 and LBP as biomarkers of sarcoidosis, in comparison with sCD25, in a cohort of well-characterized sarcoidosis patients and healthy controls. Secondary analyses within the sarcoidosis group assessed whether these biomarkers, together with ACE, were associated with specific organ involvement and examined their longitudinal changes during immunosuppressive therapy.

## 2. Materials and Methods

### 2.1. Study Population

We included patients aged >18 years with sarcoidosis diagnosed between 1 December 2014 and 28 March 2025, having consented to the Swiss SLE Cohort Study (SSCS; Swissethics 2017-01434) and for reuse of data and biological samples. The diagnosis of sarcoidosis was established either by histopathological confirmation of non-caseating granulomas or by a typical clinical presentation such as Löfgren syndrome, in accordance with established diagnostic criteria. Exclusion criteria included active tuberculosis or non-mycobacterial infection, other non-infectious granulomatous diseases (berylliosis, histiocytosis), common variable immunodeficiency, or chronic granulomatous disease.

For biological samples collected prior to inclusion in the SSCS, patients were required to have signed the general consent of the Lausanne University Hospital (Centre Hospitalier Universitaire Vaudois, CHUV), allowing the reuse of clinical data and biological material for research purposes.

All subjects were included prior to the initiation of immunomodulatory or immunosuppressive treatment.

We established a control group consisting of individuals aged >18 years without clinical or biological evidence of autoimmune or inflammatory diseases. They were recruited as part of the Swiss Immune Setpoint Study (Swissethics 2018-01932) and matched to the sarcoidosis group based on sex and age. All participants provided written informed consent. Individuals with clinically significant acute or chronic illnesses (e.g., viral hepatitis), autoimmune diseases, primary or secondary immunodeficiency (including HIV infection), recent use of medications known to modify immune function (such as corticosteroids, immunosuppressants or immunomodulators), pregnancy, or blood donation within the preceding two months were excluded.

Organ involvement was assessed at the time of the first biological sampling using the revised WASOG instrument published in 2014 [15]. Organ involvement was defined when biopsy-confirmed non-necrotizing epithelioid granulomas or when clinical manifestations met the “highly probable” or “probable” categories. Cardiac involvement was determined according to the 2016 Japanese Circulation Society (JCS) guidelines [16] or the Heart Rhythm Society 2014 consensus statement [17].

Demographic data were obtained from the SSCS database, the Swiss Immune Setpoint Study database, or extracted from patient medical records. BMI (kg/m^2^) was calculated from recorded height and weight.

### 2.2. Laboratory Measurements

Blood samples were obtained from both the research cohort and routine diagnostic collections and were processed according to the same standardized protocol (centrifugation at 1990× *g* for 10 min at 24 °C, aliquoting, and storage at −80 °C within 24 h).

To assess matrix comparability for LBP, we measured LBP in six matched plasma–serum sample pairs analyzed in parallel. Plasma and serum concentrations were strongly correlated (r = 0.90). Linear regression analysis showed a proportional bias (slope = 0.84) with a small constant offset (intercept = 4.16), indicating systematically lower values in serum compared with plasma. Given this systematic difference, all subsequent analyses were performed using serum samples only. When two samples were available for a patient, determinations for a given biomarker were performed in a single analytical run to minimize potential inter-assay variability.

Serum ACE levels were measured by kinetic assay (Bühlmann Laboratories, Switzerland) on a Roche Cobas Integra^®^ analyzer (Roche Diagnostics, Rotkreuz, Switzerlan), using the manufacturer’s reference range of 20–70 U/L. ACE was not measured in healthy controls due to insufficient remaining serum volume in the control biobank samples; therefore, ACE was not included in case–control comparisons. ACE measurements were used only for secondary analyses within the sarcoidosis group, including evaluation of organ involvement and longitudinal changes under treatment. Serum sCD14 was quantified with a commercial ELISA (Quantikine^®^ Human CD14 Immunoassay, R&D Systems/Bio-Techne; Cat. DC140; Lot P424811) according to the manufacturer’s instructions. Serum sCD25 (sIL-2Rα) was measured by sandwich ELISA (Quantikine^®^, R&D Systems/Bio-Techne; Cat. DR2A00, Lot P470209) following the manufacturer’s instructions. Serum LBP was determined using a commercial ELISA (Hycult Biotech; Cat. HK315, Lot 36519K1024-T) according to the manufacturer’s instructions. All ELISAs were performed on a Dynex Agility^®^ automated platform. According to the manufacturer’s specifications, the analytical ranges were 250–16,000 pg/mL for sCD14 and 78–5000 pg/mL for sCD25. For LBP, the analytical range was 4.4–50 ng/mL. Results were converted to ng/mL for sCD14 and to µg/mL for LBP for reporting purposes. Calibration curves were generated at each analytical run in accordance with the manufacturer’s instructions. The mean intra-assay and inter-assay coefficients of variation reported by the manufacturer were 5.5% and 6.3% for sCD14, and 2.35% and 4.1% for LBP, respectively. For sCD25, which is routinely measured in our laboratory, internal quality assessment showed mean intra-assay and inter-assay coefficients of variation of approximately 12%.

Routine laboratory parameters (CRP, ESR, hematology, renal and liver tests, calcium) were extracted when available within ±14 days of biomarker sampling. Some values were missing due to routine clinical practice. Estimated glomerular filtration rate (eGFR) was excluded because calculation methods varied over the study period.

### 2.3. Statistical Analysis

Analyses were performed using R software (version 4.5.1; R Foundation for Statistical Computing, Vienna, Austria; https://www.R-project.org/, accessed on 16 March 2026) and Stata 17.0 (StataCorp, College Station, TX, USA). Descriptive statistics summarized demographic and clinical characteristics. Continuous variables were compared between sarcoidosis patients and healthy controls using the Wilcoxon rank-sum test, while categorical variables were analyzed using chi-square as appropriate. Correlations between quantitative variables were assessed using Spearman’s rank correlation coefficient.

Receiver operating characteristic (ROC) curves were generated to evaluate the diagnostic performance of individual biomarkers (LBP, sCD25, and sCD14) in distinguishing sarcoidosis patients from healthy controls, using data from the first blood sample only. Participants with missing biomarker values were excluded from the analysis. Optimal cutoff values for each biomarker were determined using Youden’s index.

A multivariable logistic regression model with stepwise selection based on the Akaike Information Criterion (AIC) was used to identify independent factors associated with sarcoidosis. The initial model included sCD25, BMI, LBP and sCD14. Based on prior evidence of its strong association with disease activity, sCD25 (expressed per + 100 pg/mL) was pre-specified to remain in all models. The remaining candidate variables (BMI, LBP, and sCD14) were entered into the stepwise selection procedure. Odds ratios (ORs) with 95% confidence intervals (CIs) were reported. Model performance was assessed using receiver operating characteristic (ROC) curve analysis and the area under the curve (AUC). To ensure robustness, the analysis was repeated using bootstrap resampling (1000 iterations with 80/20 train–test splits), and the optimal cutoff was determined by the Youden index in each iteration. Age and sex were not included in the regression models because cases and controls were individually matched for these variables.

For each organ-specific manifestation of sarcoidosis, the discriminative ability of individual biomarkers (LBP, sCD25, sCD14, ACE) was evaluated using nonparametric ROC curve analysis. The AUC and its 95% CIs were estimated using the DeLong method and validated by stratified bootstrap resampling (1000 iterations). Organs with fewer than five positive or negative cases were excluded from analysis.

Within-patient changes in biomarker levels between baseline (T1) and follow-up (T2) samples were assessed using paired Wilcoxon signed-rank tests in patients with two available measurements.

Statistical significance was set at *p* < 0.05 (two-sided).

The power calculation was based on published data for sCD14 levels in sarcoidosis [18]. The minimum sample size of approximately 46 subjects per group (untreated sarcoidosis vs. controls) was required to detect a difference of 0.59 mg/mL with 90% power and a two-sided alpha of 0.05.

### 2.4. Ethics Approval

The present analysis was approved by the Cantonal Ethics Committee for Research Involving Human Subjects of Canton de Vaud (Swissethics, BASEC number 2025-00138). The study was conducted in accordance with the Declaration of Helsinki and in compliance with previously approved cohort protocols (Swissethics 2017-01434 and 2018-01932).

## 3. Results

### 3.1. Demographics

A total of 46 patients with sarcoidosis and 46 age- and sex-matched controls were included. Among the sarcoidosis group, 45 had biopsy-confirmed non-caseating granulomas, and 4 presented with Löfgren’s syndrome.

Baseline characteristics are summarized in Table 1. The mean age was 50 years, and each group included 28 males (61%). Sarcoidosis patients had a significantly higher BMI compared to controls (*p* = 0.003).

Among the 46 patients with sarcoidosis, 43 (93.5%) had multisystemic disease. The median number of organs involved was 3 [2–5]. The most frequent extrapulmonary sites were extra-thoracic lymph nodes (56.5%) and cardiac involvement (52.2%) (Table 2).

Of the 32 patients with sequential samples taken during immunomodulatory treatment, 19 (56%) were under dual therapy, 8 (25%) under monotherapy, and 5 (16%) under triple therapy. The median interval between blood samplings was 343 days (IQR 222–653 days). The distribution of treatment regimens at the second sampling is presented in Supplementary Table S2.

### 3.2. Biomarker Analysis

Serum concentrations of three biomarkers—LBP, sCD14 and sCD25—were compared between healthy controls and sarcoidosis patients. ACE could not be assessed in healthy controls due to insufficient available material; therefore, no case–control comparison was possible for this biomarker.

In unadjusted analyses, no significant difference in sCD14 levels was observed between controls and patients (*p* = 0.055). In contrast, sarcoidosis patients exhibited significantly higher concentrations of LBP and sCD25 compared with controls (both *p* < 0.001). After individual matching on age and sex, sCD14 levels were also significantly higher in sarcoidosis patients (*p* = 0.028) (Table 3).

Routine laboratory parameters available at baseline are summarized in Supplementary Table S1. Correlations between routine inflammatory markers and the investigated biomarkers were examined. LBP showed modest correlations with CRP (ρ = 0.41, *p* = 0.009) and ESR (ρ = 0.38, *p* = 0.013). sCD25 correlated weakly with ESR (ρ = 0.33, *p* = 0.031), whereas correlations with sCD14 and ACE were not statistically significant.

We fitted a stepwise (AIC-based) logistic regression with a binary outcome (sarcoidosis vs. control), pre-specifying sCD25 (per +100 pg/mL) to remain in all models based on prior evidence that this marker is consistently elevated and strongly associated with sarcoidosis activity. Candidate variables included BMI (kg/m^2^), LBP, and sCD14 (per +100 ng/mL). Age and sex were not included, as the two groups were matched for these variables. The final model retained BMI in addition to sCD25, while LBP and sCD14 were not selected.

Compared with the sCD25-only model, adding BMI significantly improved model fit (likelihood-ratio test χ^2^ = 4.49, *p* = 0.034; final AIC = 66.40). In the final model, sCD25 (per +100 pg/mL) remained strongly associated with sarcoidosis (OR 1.53, 95% CI 1.26–1.86, *p* < 0.001). BMI (per 1 kg/m^2^) was also independently associated with sarcoidosis (OR 1.18, 95% CI 1.01–1.38, *p* = 0.044). Model discrimination was excellent (AUC = 0.927). Youden-optimized operating points were approximately 0.56 (sensitivity 0.82; specificity 0.93) and 0.68 (sensitivity 0.80; specificity 0.96). For diagnostic confirmation (rule-in), the ~0.68 threshold is preferred (Table 4).

**Figure 1:**
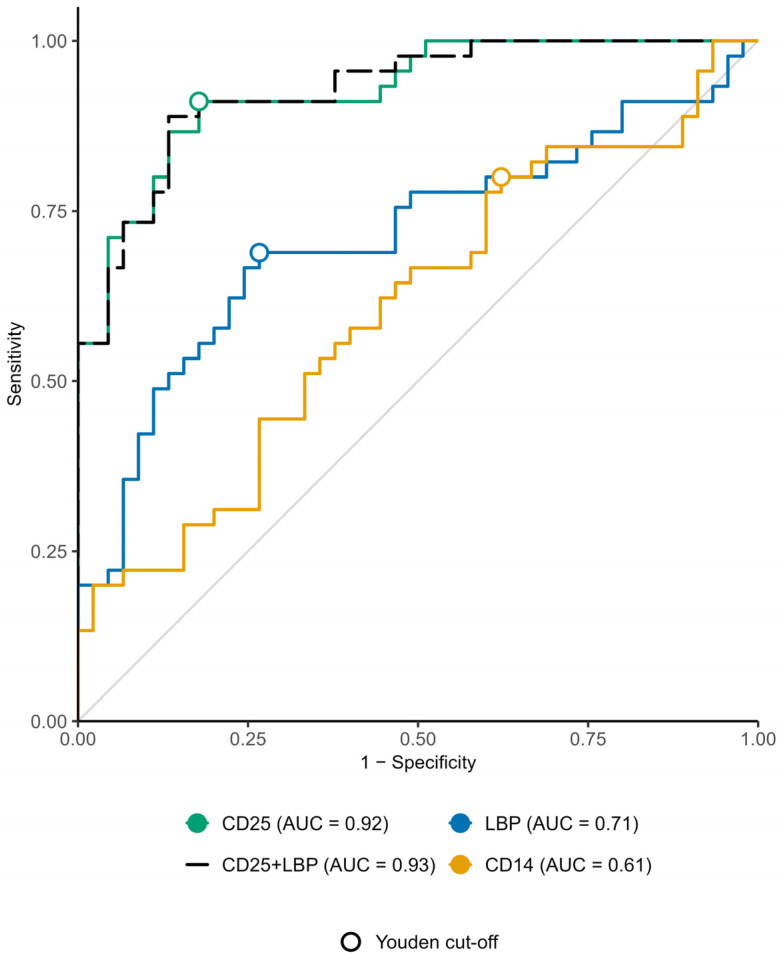
ROC curve analysis for LBP, sCD25 and sCD14.

ROC curve analyses comparing sarcoidosis patients with controls are presented in Figure 1. Among the biomarkers evaluated, sCD25 demonstrated the highest discriminatory performance, with an AUC of 0.92 (95% CI 0.87–0.98), markedly outperforming LBP (AUC 0.71, 95% CI 0.60–0.82) and sCD14 (AUC 0.61, 95% CI 0.49–0.73). At the respective optimal cut-offs (sCD25: 817.6 pg/mL, LBP: 10.71 µg/mL, sCD14: 1095 ng/mL), the operating points (red dot) corresponded to sensitivities and specificities of 91% and 82% for sCD25, 69% and 73% for LBP, and 83% and 38% for sCD14. In a multivariate logistic regression model including sCD25 and LBP, only sCD25 remained significantly associated with sarcoidosis (OR 1.56 per 100 pg/mL increase; 95% CI 1.28–1.90; *p* < 0.001), whereas LBP did not provide additional predictive value (OR 1.06 per 1 µg/mL increase; 95% CI 0.88–1.29; *p* = 0.54). These findings indicate that sCD25 alone offers superior diagnostic accuracy, and the inclusion of LBP does not significantly improve discrimination.

**Figure 2:**
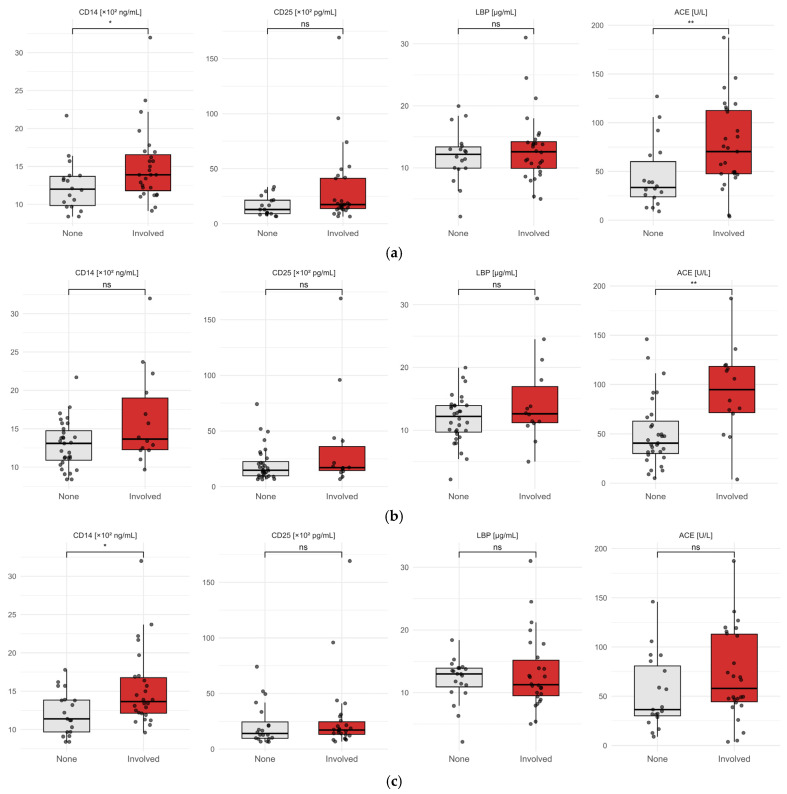
Serum biomarker levels by organ involvement. (**a**) Pulmonary involvement. (**b**) Splenic involvement. (**c**) Extra-thoracic node involvement.

In the overall sample (n = 90), biomarkers exhibited moderate positive correlations (Figure 2): sCD25 (per +100 pg/mL) with sCD14 (per +100 ng/mL) (ρ = 0.438, *p* < 0.001), sCD25 with LBP (ρ = 0.451, *p* < 0.001), and sCD14 with LBP (ρ = 0.351, *p* < 0.001). Stratified analyses suggested slightly stronger associations among sarcoidosis cases compared to controls. ACE was not included in correlation analyses involving both cases and controls, as it was measured exclusively in sarcoidosis patients.

ACE, sCD25, sCD14 and LBP levels were further assessed according to organ involvement in sarcoidosis patients. After excluding 4 patients on ACE inhibitors (n = 42), median ACE levels were significantly higher in patients with splenic involvement (105.80 U/L [IQR 83.20–128.40] vs. 42.10 U/L [IQR 24.30–59.90], *p* < 0.001) and pulmonary involvement (74.95 U/L [IQR 42.21–107.69] vs. 34.80 U/L [IQR 14.50–55.10], *p* < 0.01). Median sCD14 levels were also elevated in patients with pulmonary involvement (1420 ng/mL [IQR 1174–1666] vs. 1190 ng/mL [IQR 1004–1376], *p* = 0.02) and in those with extra-thoracic lymph node involvement (1390 ng/mL [IQR 1153–1627] vs. 1120 ng/mL [IQR 913–1326], *p* = 0.01). No significant differences were observed for sCD25 or LBP across these organ-specific subgroups in univariate analyses. Biomarker levels did not differ substantially between patients with and without other organ involvement (e.g., liver, heart, bone). For ocular, renal, cutaneous, and neurological involvement, small sample sizes precluded reliable conclusions.

To evaluate the discriminative ability of each biomarker (sCD25, sCD14, LBP, ACE) for predicting organ involvement, we calculated the AUC. For pulmonary involvement, ACE demonstrated the highest performance (AUC = 0.80; 95% CI = 0.65–0.95), followed by sCD14 (AUC = 0.71; 95% CI = 0.55–0.87). For splenic involvement, ACE achieved an AUC of 0.84 (95% CI = 0.71–0.96). For extrathoracic lymph node, sCD14 showed an AUC of 0.74 (95% CI = 0.58–0.90).

Hepatic, cardiac, skin, and bone involvement exhibited only moderate discriminative ability, with AUC values generally below 0.7, while data for other organs were inconclusive due to the limited sample size.

Among 32 patients with a second blood draw during follow-up under treatment, all biomarkers (sCD25, ACE, LBP and sCD14) showed significant decreases (Table 5). However, because of the heterogeneity of treatment regimens and the limited sample size, treatment-specific effects on biomarker dynamics could not be reliably evaluated. The distribution of treatment regimens is presented in Supplementary Table S2.

In an exploratory Poisson model assessing total organ involvement, only sCD14 retained an association (Rate Ratio 1.04, 95% CI 1.01–1.07; *p* = 0.008), but the effect size was minimal, suggesting limited clinical relevance and possibly background immune activation rather than a true marker of disease burden.

## 4. Discussion

### 4.1. Diagnostic Performance of Circulating Biomarkers

In this study, sCD25 showed the best diagnostic performance among the evaluated biomarkers, outperforming both sCD14 and LBP. Although LBP and sCD14 were modestly elevated in sarcoidosis patients, neither marker provided additional diagnostic value beyond sCD25 in multivariable analyses.

sCD25 is a well-established biomarker reflecting T-cell activation and has consistently been reported to be elevated in sarcoidosis [19,20]. Several studies have demonstrated that serum sCD25 levels are elevated in patients with sarcoidosis and may provide higher diagnostic sensitivity compared with traditional biomarkers such as ACE [7,10,20,21]. Interestingly, the optimal sCD25 cutoff identified in our cohort (817.6 pg/mL) was substantially lower than values reported in other studies. A recent meta-analysis indicates sCD25 cutoff ranges from approximately 2300 to 5800 pg/mL [6]. This discrepancy likely reflects considerable heterogeneity across studies, including differences in patient populations, disease severity, sampling timing, the choice of control group, and the analytical methods used for sCD25 quantification. Such variability underscores the challenges in standardizing and interpreting sCD25 as a diagnostic biomarker in sarcoidosis.

In contrast, the innate immune markers evaluated in this study, LBP and sCD14, showed more limited diagnostic utility. sCD14 and LBP were selected as biomarkers for sarcoidosis due to their central role in innate immune activation and granuloma formation. Both proteins are key components of the TLR4 complex, which recognizes pathogen-associated molecular patterns (PAMPs) and triggers pro-inflammatory cytokine release [22].

In our study, sCD14 levels were significantly higher in sarcoidosis patients after individual matching on age and sex. Previous reports have described elevated CD14 concentrations in sarcoidosis [13,18]. Striz et al. demonstrated that sCD14 is increased in bronchoalveolar lavage of patients with active sarcoidosis and correlates with membrane-bound CD14 expression on alveolar macrophages [23]. However, another study did not find any significant difference in serum sCD14 levels between sarcoidosis patients and controls, highlighting the need for caution in interpreting these results [11]. Notably, while earlier studies predominantly enrolled patients with isolated pulmonary disease [13,18], our cohort consisted almost entirely of multisystemic cases (93.5%). The discrepancy with the results from Futami et al., whose cohort also included multisystemic cases, might be better explained by differences in statistical adjustment for demographic factors. In line with this, our unadjusted analysis showed no significant difference (*p* = 0.055) before individual matching for age and sex.

Mechanistically, LBP occupies a pivotal position in innate immune activation, binding pathogen-associated molecular patterns (PAMPs) and facilitating downstream signaling [14]—a pathway implicated in sarcoidosis pathogenesis [22]. Importantly, its role in sarcoidosis remains underexplored; to date, only one prior study has specifically addressed this question [11]. These findings highlight the need for larger prospective investigations to clarify whether LBP represents a meaningful biomarker or a mechanistic epiphenomenon.

Furthermore, the relevance of biological compartmentalization is underscored: sCD14 and LBP appear highly enriched within granulomatous tissue or at the cellular level, yet their circulating concentrations may be attenuated by dilution. Consistently with this, Futami et al. demonstrated robust CD14 and LBP expression in granulomatous lesions, suggesting these molecules may hold greater promise as tissue-based or extracellular vesicle biomarkers rather than as circulating screening tools [11]. Proteomic approaches can identify molecules involved in disease pathophysiology even when their circulating concentrations remain too low or variable to provide reliable diagnostic discrimination [24]. In addition, LBP and sCD14 capture upstream innate immune activation related to triggering events, whereas sCD25 better reflects the downstream immune response sustaining clinically overt sarcoidosis. This may explain the superior discriminative performance of sCD25 at the time of diagnosis.

BMI was included as a covariate in the multivariable models because adipose tissue plays a role in modulating systemic inflammation through the secretion of cytokines and adipokines. This adjustment is important as adiposity may influence circulating immune biomarkers and potentially confound their association with sarcoidosis [25,26]. Interestingly, BMI was independently associated with sarcoidosis in multivariable analysis. Prior evidence on this relationship remains inconsistent: some studies have reported an association, particularly among African American women in the United States [27,28], whereas a large retrospective analysis in male veterans found no such link [29]. Although the connection between overweight and sarcoidosis is still debated, obesity is increasingly recognized as an immunometabolic state characterized by chronic low-grade inflammation mediated by adipose-derived cytokines [30]. Consequently, BMI or adiposity-related markers may act as confounders or effect modifiers in biomarker research and should be systematically considered in future studies. Notably, BMI has inherent limitations, as it does not differentiate adipose tissue from lean mass. Future investigations should therefore incorporate body composition measures and/or adipokine and myokine profiling.

We further examined the utility of serum biomarkers for predicting organ involvement. In our study, ACE demonstrated the highest discriminative ability for pulmonary involvement, aligning with Zhou et al. [31]. However, its discriminative ability for pulmonary involvement is modest, as elevations are also seen in multisystemic sarcoidosis and other conditions [3,31,32]. This association is driven by the near-universal presence of pulmonary disease in these cohorts. ACE remains non-specific and is influenced by genetic polymorphisms and the use of ACE inhibitors [33,34,35]. It reflects overall granulomatous burden, produced by activated alveolar macrophages, rather than organ-specific disease activity [34].

The association of serum sCD14 with pulmonary and nodal involvement aligns with reports linking this marker to macrophage activation and respiratory function impairment [13,18,23]. However, its modest predictive utility suggests that circulating sCD14 reflects systemic innate immune activation that is likely diluted in serum.

sCD25 did not correlate with the number or localisation of involved organs in our cohort, despite prior reports indicating higher concentrations in multisystemic sarcoidosis [31,36]. This lack of correlation may be due to the systemic nature of sCD25, reflecting overall immune activation rather than specific organ involvement [37]. Accurately quantifying granulomatous burden remains challenging and will likely require imaging-based approaches, such as PET-derived algorithms.

### 4.2. Longitudinal Changes Under Immunosuppressive Therapy

All four biomarkers (sCD25, sCD14, LBP and ACE) significantly decreased under immunosuppressive therapy. This finding suggests that these markers may reflect treatment-related changes and could potentially serve as pharmacodynamic indicators. However, this decrease should be interpreted with caution, as it may partly reflect a general reduction in systemic inflammation rather than a sarcoidosis-specific change in disease activity. Consistent with this interpretation, LBP showed moderate correlations with CRP and ESR in our cohort. The utility of these biomarkers in guiding treatment decisions therefore warrants further prospective evaluation.

### 4.3. Strengths and Limitations

Key strengths include treatment-naïve patients from a well-characterized sarcoidosis cohort and a matched case–control design allowing assessment of diagnostic performance under real-world conditions.

However, several limitations should be acknowledged. First, the single-center design and relatively small sample size may limit the generalizability of our findings. Second, as a retrospective analysis, organ involvement was determined through clinically driven assessments rather than systematic multi-organ screening, which may have led to underdetection of subclinical manifestations, particularly in organs such as the eye, kidney, heart, or nervous system. Routine laboratory parameters were not systematically available for all patients, as these tests were performed as part of standard clinical care. FDG-PET imaging was not uniformly performed, and in some cases, a time interval existed between imaging and blood sampling, potentially introducing temporal discordance between biomarker levels and visible metabolic activity. In addition, ACE was not measured in the control group, precluding direct case–control comparison for this marker. Small sample sizes for rarer manifestations (e.g., ocular, renal, and neurological involvement) limited our ability to establish organ-specific correlations in those domains. A standardized disease activity score was not available for this retrospective cohort. Currently, no universally accepted activity score exists for sarcoidosis, and assessment of disease activity typically relies on a multidimensional evaluation combining clinical, radiological and biological parameters. Several attempts have been made to develop imaging-based activity scores, but these are not yet widely implemented in routine clinical practice [38]. Moreover, both sCD14 and LBP reflect activation of the innate immune system and are not disease-specific markers. Their circulating levels may therefore be influenced by other inflammatory or metabolic conditions, which should be considered when interpreting the results. These findings therefore require confirmation in larger independent cohorts to allow external validation.

## 5. Conclusions

In this matched case–control study, sCD25 emerged as the most robust serum biomarker for sarcoidosis diagnosis, outperforming sCD14 and LBP and remaining independently associated with disease status in multivariable models. These findings reinforce the diagnostic value of sCD25, while underscoring the need for validation in larger multicenter cohorts and its integration with emerging immunometabolic biomarkers.

## Figures and Tables

**Table 1:** Demographic data in controls and in patients with sarcoidosis.

Variables	Control (n = 46)	Sarcoidosis (n = 46)	*p*-Value
Male sex, n (%)	28 (60.9)	28 (60.9)	
Caucasian, n (%)		42 (91.3)	
Age, mean (SD)	50 (13.0)	50 (13.1)	
Weight, mean (SD)	72.6 (13.7)	80.7 (19.4)	0.029
Height, mean (SD)	1.73 (0.09)	1.71 (0.09)	0.243
Body mass index, kg/m^2^, mean (SD)	24.2 (3.5)	27.7 (6.8)	0.004
Former or active smoker, n (%)		12 (26)	
ACE inhibitor use, n (%)		4 (8.7)	

**Table 2:** Organ involvement in patients with sarcoidosis (n = 46).

Variables	Sarcoidosis (n = 46)
Isolated pulmonary disease	3 (6.5)
Multisystemic sarcoidosis	43 (93.5)
Type of extra-thoracic involvement	
Extra-thoracic lymph nodes	26 (56.5)
Heart	24 (52.2)
Spleen	14 (30.4)
Liver	11 (23.9)
Bone	11 (23.9)
Skin	9 (19.6)
Nervous system	5 (10.9)
Eye	3 (6.5)
Kidney	2 (4.3)
Number of organs involved, median [IQR]	3 [2–5]

Data are presented as n (%) or median [IQR]; isolated pulmonary disease was defined as pulmonary parenchymal disease and/or intrathoracic (hilar/mediastinal) lymph node involvement without extrapulmonary organ involvement. For organ counts, pulmonary parenchyma and intrathoracic lymph nodes were considered a single organ.

**Table 3:** Biochemical parameters in age- and sex-matched controls and patients with sarcoidosis.

Variables	Control	Sarcoidosis	*p*-Value
	**(n = 46)**	**(n = 46)**	
LBP (µg/mL)	9.7 [8.3–10.8]	12.5 [10.0–13.9]	<0.001
sCD14 (ng/mL)	1180 [1010–1452.5]	1320 [1120.0–1570.0]	0.028
sCD25 (pg/mL)	640.2 [557.9–763.6]	1666.3 [1071.3–2454.9]	<0.001

Data are presented as median [IQR]; lipopolysaccharide-binding protein (LBP), soluble CD14 (sCD14), soluble interleukin-2 receptor alpha chain (sCD25).

**Table 4:** Stepwise (AIC) logistic regression model for sarcoidosis vs. controls.

Variables	*p*-Value	Odds Ratio	95% Confidence Interval
sCD25 (per 100 pg/mL)	<0.001	1.53	1.258–1.862
BMI (per 1 kg/m^2^)	0.044	1.179	1.005–1.383

sCD25 forced during stepwise.

**Table 5:** Within-patient changes in biomarkers between baseline (T1) and follow-up (T2).

Biomarker (Units)	n Pairs	T1 Median [IQR]	T2 Median [IQR]	Wilcoxon V	*p*-Value
sCD25 (pg/mL)	32	1620.5 [945.1–2349.5]	978.7 [689.5–1299.2]	497.0	<0.0001
sCD14 (ng/mL)	31	1380.0 [1135.0–1570.0]	1160.0 [1040.0–1245.0]	458.5	<0.0001
LBP (µg/mL)	32	12.8 [10.8–14.0]	10.7 [8.6–12.3]	432.0	0.0017
ACE (U/L)	32	48.4 [34.2–84.1]	33.0 [23.9–42.4]	438.5	0.0011

## Data Availability

The datasets generated and analyzed during the current study are available in the Zenodo repository (https://doi.org/10.5281/zenodo.18722377, accessed on 16 March 2026). Due to ethical and data protection regulations involving clinical patient data, access to the files is restricted and granted upon reasonable request and institutional approval.

## Supplementary Materials

The following supporting information can be downloaded at: https://www.mdpi.com/article/10.3390/diagnostics16071018/s1, Table S1: Routine laboratory parameters in sarcoidosis patients at baseline, Table S2: Treatment regimens at the time of second sampling in 32 patients with sarcoidosis, Table S3: Diagnostic performance of evaluated biomarkers for distinguishing sarcoidosis patients from healthy controls.

## Abbreviations

The following abbreviations are used in this manuscript:

ACE	Angiotensin-Converting Enzyme
LBP	Lipopolysaccharide-binding protein
sCD25	soluble interleukin-2 receptor (sCD25)
CD14	Cluster of Differentiation 14
BMI	Body Mass Index
FDG-PET	18F-Fluorodeoxyglucose Positron Emission Tomography
PAMPs	Pathogen-Associated Molecular Patterns
WASOG	World Association of Sarcoidosis and Other Granulomatous Disorders
JCS	Japanese Circulation Society
