# Supplementary material for: Soluble CD14 and Lipopolysaccharide-Binding Protein Are Not Superior to Soluble CD25 as Biomarkers for Sarcoidosis
Source: Diagnostics (Basel). 2026 Mar 28;16(7):1018. doi: 10.3390/diagnostics16071018 (PMC13073008; doi:10.3390/diagnostics16071018)
Supplement: Supplementary file 1 [file diagnostics-16-01018-s001.zip › diagnostics-4192912-supplementary.pdf]

diagnostics

MDPI

Table S1. Routine laboratory parameters in sarcoidosis patients at baseline

Parameter	n	Value
CRP (mg/L)	39	3.1 [2–7.1]
ESR (mm/h)	42	12.5 [7–20]
Hemoglobin (g/L)	46	143 [133.25–151]
Leukocytes (G/L)	46	6.8 [5.3–8.4]
Platelets (G/L)	46	262 [219–288.75]
Creatinine (µmol/L)	45	86 [73–98]
AST (U/L)	41	24 [20–34]
ALT (U/L)	42	30.5 [22–43.75]
ALP (U/L)	37	76 [63–93]
GGT (U/L)	36	36.5 [27.25–62]
Calcium (mmol/L)	36	2.34 [2.29–2.42]

* Data are presented as median [IQR]. Laboratory values were extracted when available within a ±14-day window around the first biomarker sampling. CRP, C-reactive protein; ESR, erythrocyte sedimentation rate; AST, aspartate aminotransferase; ALT, alanine aminotransferase; ALP, alkaline phosphatase; GGT, gamma-glutamyl transferase.

Table S2. Treatment regimens at the time of second sampling in 32 patients with sarcoidosis

Regimen	N (%)
By regimen class (any use within the regimen)	
Prednisone containing regimen	21 (66)
Classical immunosuppressants	17 (53)
Antimalarials	12 (38)
TNF blocker	11 (34)
By individual agent (any use, across combinations)	
Prednisone	21 (66)
Methotrexate	15 (47)
Hydroxychloroquine	12 (38)
Infliximab	7 (22))
Adalimumab	4 (13)
Azathioprine	1 (3)
Mycophenolate mofetil	1 (3)

* Classical immunosuppressants include methotrexate, azathioprine and mycophenolate mofetil.

Table S3. Diagnostic performance of evaluated biomarkers for distinguishing sarcoidosis patients from healthy controls

Biomarker	AUC (95% CI)	Optimal cutoff	Sensitivity (%)	Specificity (%)
sCD25	0.92 (0.87–0.98)	817.6 pg/mL	91	82
LBP	0.71 (0.60–0.82)	10.71 µg/mL	69	73
sCD14	0.61 (0.49–0.73)	1095 ng/mL	83	38

*Optimal cut-off values were determined using the Youden index. sCD25, soluble interleukin-2 receptor alpha chain; sCD14, soluble CD14; LBP, lipopolysaccharide-binding protein.
